# Intrinsic Indicator of Photodamage during Label-Free Multiphoton Microscopy of Cells and Tissues

**DOI:** 10.1371/journal.pone.0110295

**Published:** 2014-10-24

**Authors:** Roberta Galli, Ortrud Uckermann, Elisabeth F. Andresen, Kathrin D. Geiger, Edmund Koch, Gabriele Schackert, Gerald Steiner, Matthias Kirsch

**Affiliations:** 1 Clinical Sensoring and Monitoring, Anesthesiology and Intensive Care Medicine, Faculty of Medicine Carl Gustav Carus, TU Dresden, Dresden, Germany; 2 Neurosurgery, Carl Gustav Carus University Hospital, TU Dresden, Dresden, Germany; 3 Neuropathology, Institute for Pathology, Carl Gustav Carus University Hospital, TU Dresden, Dresden, Germany; 4 CRTD/DFG-Center for Regenerative Therapies Dresden - Cluster of Excellence, Dresden, Germany; University of Zurich, Switzerland

## Abstract

Multiphoton imaging has evolved as an indispensable tool in cell biology and holds prospects for clinical applications. When addressing endogenous signals such as coherent anti-Stokes Raman scattering (CARS) or second harmonic generation, it requires intense laser irradiation that may cause photodamage. We report that increasing endogenous fluorescence signal upon multiphoton imaging constitutes a marker of photodamage. The effect was studied on mouse brain in vivo and ex vivo, on ex vivo human brain tissue samples, as well as on glioblastoma cells in vitro, demonstrating that this phenomenon is common to a variety of different systems, both ex vivo and in vivo. CARS microscopy and vibrational spectroscopy were used to analyze the photodamage. The development of a standard easy-to-use model that employs rehydrated cryosections allowed the characterization of the irradiation-induced fluorescence and related it to nonlinear photodamage. In conclusion, the monitoring of endogenous two-photon excited fluorescence during label-free multiphoton microscopy enables to estimate damage thresholds ex vivo as well as detect photodamage during in vivo experiments.

## Introduction

Multiphoton microscopy comprises a series of techniques that enable the investigation of native cells and tissue without the need for any labeling [1]. Two-photon excited fluorescence (TPEF) addresses endogenous fluorophores of the tissue [2]–[6], second harmonic generation (SHG) probes highly ordered structures lacking of inversion symmetry, like collagen [2], [7]–[9]. Recently, coherent anti-Stokes Raman scattering (CARS) microscopy became available to medicine, enabling the visualization of the distribution of specific molecular bonds in the tissue and proving to be very effective for imaging of lipid distribution in cells and tissue [2], [10], [11].

In order to efficiently excite endogenous nonlinear processes, a very high optical peak power is necessary [12]. For this reason, multiphoton techniques use ultrashort pulsed laser sources with near infrared emission [4]. Typically, both femtosecond and picosecond pulsed lasers are used for TPEF and SGH imaging [12], while for CARS imaging picosecond laser sources are preferred [10]. Common feature to all multiphoton microscopic techniques is also the use of high numerical aperture objectives [10], [13], to obtain a high concentration of the laser power inside the sample. In consequence of the high laser irradiance, the photodamage of cells and tissue is a still open issue. Moreover, these techniques draw increasing attention for in vivo use, where their intrinsic characteristics – omitting labels or dyes, and confocality – are better exploited.

It is widely accepted that it is possible to perform multiphoton imaging of tissue with a laser power below the damage threshold. Multiphoton microscopy with fluorescent labels is normally performed at laser power below 10 mW, which is considered to be in a safe range for tissue integrity [4], [12], [14]. Nevertheless, the efficient excitation of endogenous processes like CARS needs higher laser power [2], [10], [15]–[18]. Moreover measurements often require multiple scanning of the sample, e.g. for monitoring of dynamic processes over time, setting measurement parameters or reducing noise by image averaging, while for 3-D reconstruction the illumination intensity has to be increased up to one order of magnitude in order to collect adequate images deep in the tissue [4]. Under such kind of experimental conditions high laser irradiance is used and therefore the possibility to produce photodamage cannot be ruled out.

Photodamage produced by multiphoton microscopy has been already addressed by several research groups in different biological systems [14], [19]–[23]. The physical damage that can be produced by irradiation with ultrashort lasers was even used to perform micro- and nano-surgery on cells and tissue [24]–[27]. The relationship between photodamage and laser parameters in unstained cells revealed a two-photon excitation process as source of the damage [28]. An increase in background fluorescence associated to photodamage was already observed [29]–[31] and monitored as indication of photodamage of living neurons [32], [33]. The increase of endogenous fluorescence observed in living cells was associated to increased production of NADH [34], [35].

Here we focus on study of photodamage-induced fluorescence during label-free multiphoton microscopy of nervous tissue ex vivo and in vivo. The full characterization of photodamage induced by picosecond laser irradiation of brain tissue provided evidence that a common mechanism leading to increase of endogenous TPEF exists in many different tissue types, in vitro and in vivo, and that this mechanism is different from the physiological response of cells. CARS microscopy and vibrational spectroscopy were used to analyze the biochemical changes associated to increased TPEF signal in the irradiated tissue.

## Material and Methods

### Ethics statement

All animal experiments were performed in accordance with the guidelines of the Dresden University of Technology based on national laws that are in full agreement with the European Union directive on animal experimentation. They were approved by the animal welfare committee of Saxony, Germany (Regierungspräsidium Dresden, AZ: 24-9168.11-1/2011-39). Surgical procedures were performed under ketamine–xylazine anesthesia, and all efforts were made to minimize suffering. The mice were sacrificed by cervical dislocation.

Human tissue samples were obtained during brain surgeries. All patients gave written consent, and the study was approved by the ethics committee at Dresden University Hospital (EK 323122008).

### Animals

Experiments were performed on female nude mice NMRI ^nu/nu^ (Experimental center of the University Hospital Dresden, Germany) aged six to eight weeks. Animals were kept under pathogen-free conditions in a 12 h: 12 h light-dark cycle and received food and water ad libitum.

### In vivo imaging

The mice (n = 4) were anesthetized by intraperitoenal injection of ketamine/xylazine and a cranial window was installed. After midline scalp incision the skull was opened using a dental drill in circular area of ∼4 mm diameter. The dura was carefully removed. An in-house built device was used to avoid movement of the mouse head during the experiments. A small holder was glued to the skull using dental cement. A 4 mm large window in the holder left the cortex optically accessible. The holder was then screwed on a plate mounted on the microscope table. During in vivo multiphoton imaging a glass coverslip was placed on top of the exposed brain.

### Primary glioblastoma cell culture

Fresh human glioblastoma biopsy samples were placed immediately in Amniochrome PRO medium (Lonza, Verviers, Belgium) including 100 U/ml penicillin and 0.1 mg/ml streptomycin. After mechanical dissociation the cells were transferred into T25 cell culture flasks. Half of the medium was exchanged after 24 h; afterwards medium was exchanged as necessary. For imaging experiments, cells were transferred to glass coverslips and grown for three days.

### Sample preparation

Brain tissue and mouse organs were embedded in tissue freezing medium (Leica, Nussloch, Germany), snap frozen on dry ice and stored at −80°C. Subsequently, sections of 16 µm thickness were prepared on glass slides. Cryosections foreseen for Raman and Fourier-Transform Infrared (FT-IR) spectroscopy were prepared on low-fluorescence calcium fluoride slides. Unfixed cryosections were used in all photodamage experiments. A drop of a. dest was placed directly on the tissue section 5 min before the start of the experiment and no coverslip was used. After photodamage experiments the sections were fixed in methanol-acetone (1∶1) at −20°C for 10 minutes and stored at −20°C until staining.

### Multiphoton microscopy

The multiphoton microscope is a multimodal system with near-infrared picosecond fiber lasers, which enables the simultaneous acquisition of up to four spectrally separated signals.

The optical microscope is an upright Axio Examiner Z.1 coupled to a laser scanning module LSM 7 (all from Carl Zeiss AG, Jena, Germany) and equipped with non-descanned detectors. The excitation for TPEF and SHG is provided by an Erbium fiber laser (Femto Fiber pro NIR from Toptica Photonics AG, Munich, Germany) emitting at 781 nm with pulse length of 1.2 ps and maximum nominal power of 100 mW. The TPEF signal in the spectral range 500–550 nm was acquired in reflection. The SHG signal was acquired in transmission mode on sections and in reflection mode in vivo with band pass (BP) filter centered at 390 nm and bandwidth of 18 nm.

In order to excite the CARS signal, a second laser source was used. This source (Femto Fiber pro TNIR from Toptica Photonics AG) is tunable in the range 850–1100 nm and has a pulse length of 0.8 ps. In all CARS experiments the wavelength was set to 1005 nm (emitted power 1.5 mW), in order to resonantly excite the symmetric stretching vibration of methylene groups at 2850 cm^−1^. The CARS signal was collected in forward (in case of thin cryosections) or backward direction (in vitro, ex vivo and in vivo experiments) and filtered using a BP filter centered on 647 nm with bandwidth of 57 nm. CARS, TPEF and SHG were simultaneously excited and acquired to build multimodal images (red: CARS; green: TPEF; blue: SHG).

The excitation light was focused with a C-Apochromat 32×/0.85 objective; a 40×/0.75 W N-Achroplan dipping objective was used for imaging of cells. The laser power under the microscope was measured without objective using a power meter head (818-SL Newport Corp., Irvine, USA) connected to a current meter. The power inside the sample was then calculated using the transmission of the objective at the emission wavelengths of the lasers according to manufacturer's information (C-Apochromat 32×/0.85: ∼80% at 780 nm and ∼75% at 1005 nm; W N-Achroplan 40×/0.75: ∼80% at 780 nm and ∼72% at 1005 nm).

### Fluorescence microscopy

Additionally, endogenous fluorescence in the green spectral range was detected with a mercury lamp and suited filtering (excitation filter BP 470/40, emission filter BP 525/50), and recorded with AxioCam Mrc.

### Induction and evaluation of photodamage

In all experiments, an area of 81.3×203.9 µm^2^ was defined on the sample surface and scanned repeatedly to induce photodamage. A time series was recorded during repeated scanning. A pixel size of 0.45 µm and a pixel dwell of 9 µs were used. The acquisition time for one image was 850 ms. After the end of the serial scanning, an overview image was acquired including also the surrounding undamaged tissue areas. The TPEF pixel intensity was measured in the irradiated area during the whole time course of scanning using the open source software Fiji [36] (http://fiji.sc/Fiji). The central part of the scanned area was chosen for quantification and divided into 13 regions of interest (ROIs). Basal TPEF was calculated by averaging 10 intensity values and the onset of damage was defined by an increase in TPEF intensity of 25% above this baseline in the region of interest (Fig. S1). The number of scans that was needed to produce damage was determined for each ROI separately to account for local differences. An average threshold for all ROIs was calculated for each investigated area.

### Histology and immunohistochemistry

To visualize tissue properties, the sections were stained with hematoxylin and eosin (H&E).

Sections were washed in a. dest and incubated in Meyer's hematoxylin/hemalum (Sigma Aldrich, Steinheim, Germany) for 3 min. After washing in a. dest the tissue was briefly destained in HCl-ethanol. Washing using tap water for 5 min was followed by 3 min staining in eosin (1% eosin G in 80% ethanol). The sections were dehydrated with rising ethanol concentrations, cleared in xylene and coverslipped using DePex. For immunohistochemistry, sections were fixed in Methanol-Acetone at −20°C for 10 min followed by heat antigen retrieval in citrate buffer. After blocking in 5% bovine serum albumine in 0.3% TritonX for 1 h the tissue was probed with the primary antibody for 1 h (Anti-GFAP-Cy3, 1∶800, Sigma Aldrich). After washing with PBS, the sections were mounted with Vectashield containing DAPI (Vector Laboratories Inc., Burlingame, CA, USA).

### Transmission Electron Microscopy

After induction of photodamage, formalin fixed bulk tissues samples were fixed in 2% glutaraldehyde, embedded in epon and fixed with osmium tetroxide. The location of photodamage was determined on serial semi-thin sections of 1 µm thickness stained with tolouidine blue. From these areas 60–80 nm thick sections were cut and contrasted on grids with uranylacetate and lead citrate, and visualized with a Zeiss EM 900 transmission electron microscope.

### Vibrational spectroscopy

Raman spectra were obtained using a Raman spectrometer (RamanRxn1, Kaiser Optical Systems Inc., Ann Arbor, USA) coupled to a light microscope (DM2500 P, Leica Microsystems GmbH, Wetzlar, Germany). The excitation of Raman scattering was obtained with a diode laser emitting at a wavelength of 785 nm, propagated to the microscope with a 100 µm optical fiber and focused on the samples by means of a 50×/0.75 microscope objective, leading to a focal spot with diameter of 20 µm. The Raman signal was collected in reflection configuration and sent to the f/1.8 holographic imaging spectrograph by using a 62.5 µm core optical fiber. The spectral resolution in the range 150–3250 cm^−1^ was 4 cm^−1^. An integration time of 2 s and 10 accumulations were used for spectrum acquisition. Raman maps were recorded by a serial acquisition of spectra moving the sample on an x-y micrometer stage with a step of 25 µm. Raman data were analyzed with MATLAB workpackages (MathWorks Inc., Natick, USA). The baseline was calculated for each spectrum by applying the function “msbackadj”. Subsequent normalization of the set of spectra was obtained by standardizing the area under the curve to the group median value by using the function “msnorm”. Both functions are comprised in the Bioinformatics Toolbox. For map building, an unsupervised cluster analysis with Euclidean metric was used to group the spectra according to common spectral characteristics. The function “kmeans” of the Statistical Toolbox was used. The cluster membership information was plotted as a color-coded map.

Infrared absorption spectra were recorded with a Tensor 27 FT-IR spectrometer equipped with a microscope Hyperion 2000 (both from Bruker Optik GmbH, Ettlingen, Germany), x-y moving stage and 15× infrared objective. Detection of the signal was performed with a liquid nitrogen cooled MCT detector with KBr window. The spectral resolution was 4 cm^−1^ in the range 900–3500 cm^−1^. The lateral resolution was set to 25 µm by use of a variable square aperture. The spectra were acquired with acquisition time of 60 s. The baseline of spectra was performed with rubber band method and afterwards the spectra normalized to the total area using OPUS software of the instrument. Further analysis was performed using MATLAB.

## Results

**Figure 1:**
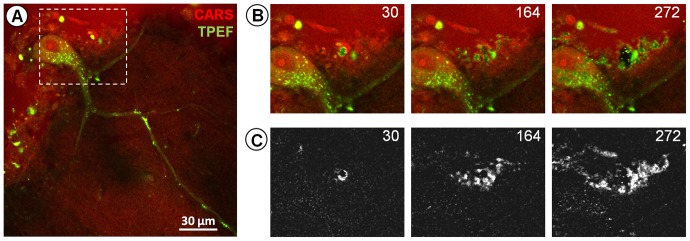
Effects of photodamage on ex vivo human cerebellum tissue during label-free multiphoton imaging. **A**: Single high quality image of a Purkinje cell acquired using CARS (red) and TPEF (green) at laser excitation power ∼52 mW. **B**: Further serial scanning of the area indicated by the dotted-line box in A resulted in a gradual increase in TPEF. The cell body is shown after 30, 164 and 272 scans. The photodamage-induced TPEF developed first in the tissue around the cell body and then also inside the cell cytoplasm. **C**: TPEF difference images (corresponding to the images in B) obtained subtracting the TPEF image of the first scan from the TPEF images at 30, 164 and 272 scans respectively; they underline the photodamage-induced TPEF around the cell (see scan 164) and in the cell cytoplasm (see scan 272).

Label-free multiphoton imaging allows the assessment of brain tissue structure and reveals cytoarchitectures. Figure 1A shows an image of a Purkinje cell in an unstained bulk sample of human cerebellum. Endogenous fluorophores within the cytoplasm can be addressed by the acquisition of TPEF (green) and CARS (red) imaging delivers morphochemical information. Normal imaging conditions using near infrared picosecond lasers and a laser power of ∼52 mW inside the sample allowed acquisition of high resolution images showing fine morphological details.

To investigate potential photodamage, we performed additional repeated scanning on the same area like required for temporally resolved imaging or the acquisition of z-stacks.

This produced an explicit increase in endogenous fluorescence detected by TPEF (Fig. 1B, C). The first indication of increased endogenous TPEF was observed in the lipid-rich tissue around the cell body after 30 additional scans. In the last stage of irradiation the increase of TPEF also included the cell cytoplasm, while the cell nucleus did not display any increase of endogenous fluorescence above background.

**Figure 2:**
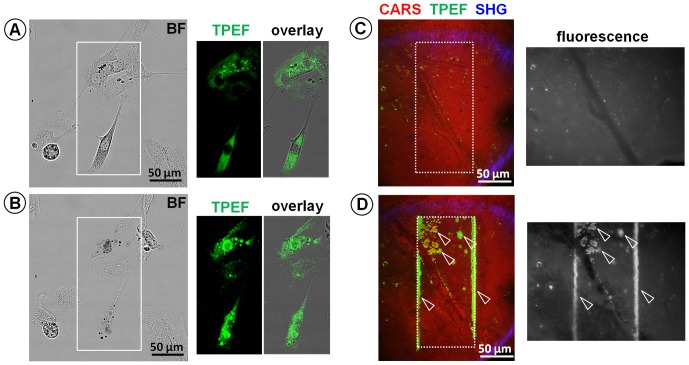
Effects of photodamage on living organisms during label-free multiphoton imaging. **A**: Bright field (BF) and TPEF image of the area in the box in the BF image acquired on living primary human glioblastoma cells in culture, and overly of BF and TPEF images. **B**: effects of a serial irradiation of 500 scans at laser power of ∼52 mW in the area indicated by the box, leading to increase of TPEF in the cell cytoplasm. C: Label-free multiphoton image and fluorescence image (acquired using a mercury lamp for excitation) of mouse brain cortex; the brain surface is slightly convex, so that the image cuts through different tissue layers: CARS shows the cortical tissue with a blood vessel, while SHG shows the inner meningeal layer on top of the cortex. D: Label-free multiphoton image and fluorescence image acquired in the same region shown in C after 544 scans performed in vivo in the area indicated by the dotted line box; the arrows indicate the localized increase of fluorescence that matches the increase of TPEF.

The same effect was observed on vital cells and in the living organism. Cultured primary glioblastoma cells (Fig. 2A, B) and the exposed cortex of living mice (Fig. 2C, D and Video S1, showing the effects of 5 minutes of serial scanning) were serially irradiated. In both cases, a gradual increase in endogenous TPEF intensity above background was observed during the time course of the experiment. The irradiation-induced fluorescence was also detected using a mercury lamp for excitation (Fig. 2D) suggesting the formation of fluorescent compounds.

**Figure 3:**
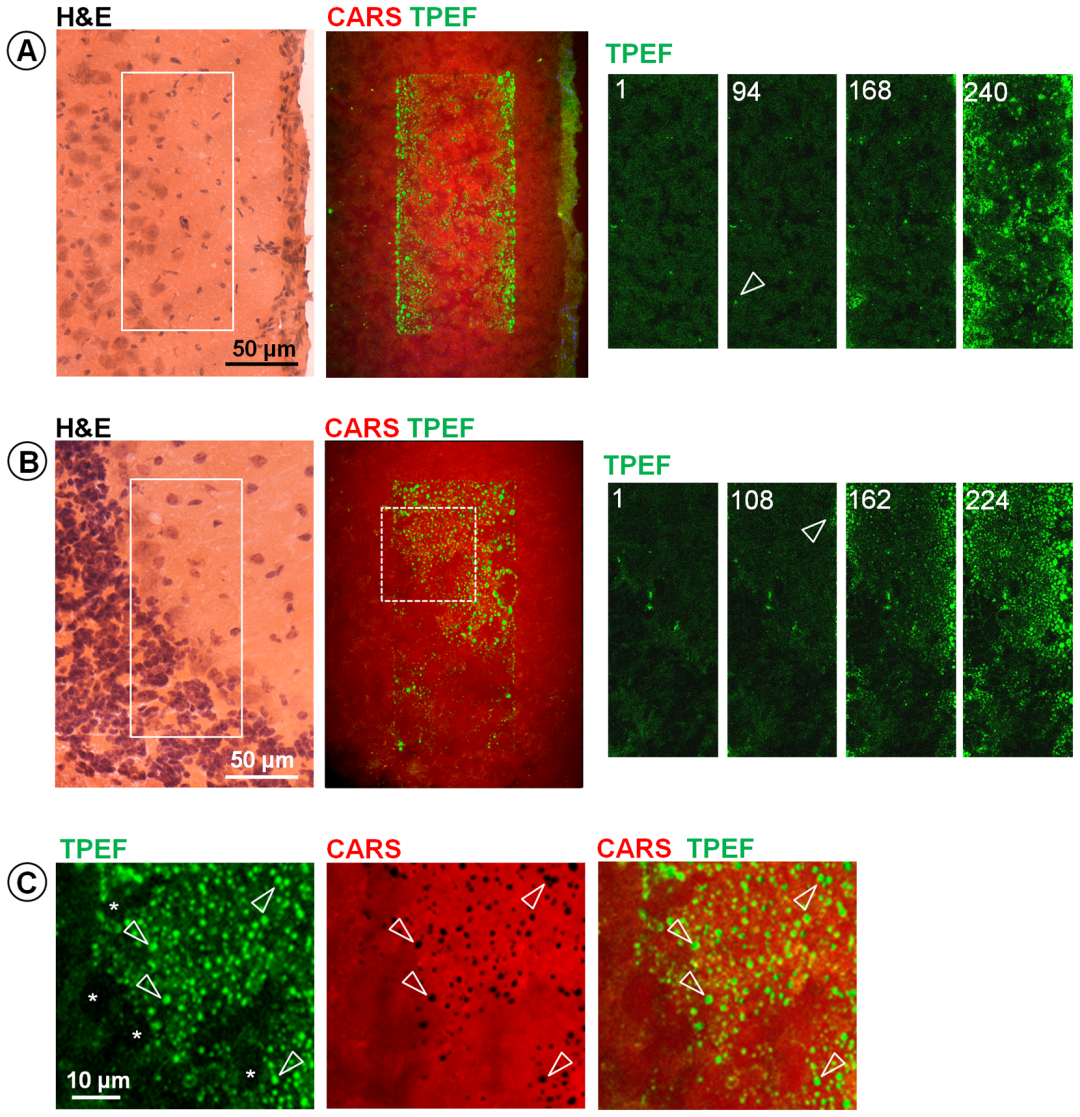
Endogenous fluorescence induced by multiphoton microscopy on rehydrated cryosections of mouse brain. **A**: Multiphoton image of cortical tissue after 240 scans in the area indicated by the box and TPEF images showing the irradiated area at scan n. 1, 94, 168 and 240; the arrow indicates the very first increase of TPEF at scan 110. **B**: Cerebellar tissue after 224 scans in the area indicated by the box and TPEF images showing the irradiated area at scan n. 1, 108, 162 and 224; the arrow indicates the very first increase of TPEF at scan n. 108. In both cases H&E staining of a consecutive section is provided as reference for tissue structures. **C**: Magnified image of the region indicated by the dashed box in B: TPEF (green) and CARS (red) are shown separately and merged. Arrows indicate spots of strong TPEF that colocalize with decreased CARS signal intensity. All experiments were performed with a total laser power of ∼52 mW in the sample.

We embarked upon exploitation of this phenomenon as a potential analytic tool. Therefore, an in vitro system was developed for characterization of irradiation-induced endogenous fluorescence. Initially, rehydrated brain cryosections were investigated because they are available in large number and enable consecutive analysis of the irradiated area with precise spatial correlation. The pattern of irradiation-induced TPEF in this system was qualitatively and quantitatively comparable to that seen in vivo. During repetitive scanning on cortical and cerebellar tissue an irregular pattern of fluorescent spots appeared in the irradiated area (Fig. 3A and B); the number and the size of these spots increased during irradiation (Video S2, showing the increase of TPEF during 10 minutes of serial scanning on cortical tissue). This increase of endogenous fluorescence was related to photochemical modifications of the tissue composition by CARS imaging, resonantly exciting the symmetric stretching vibration of CH_2_ groups at 2850 cm^−1^ to visualize lipids [11]. While TPEF was used to monitor the progressive formation of fluorescent compounds, a local decline in lipids was detected by CARS imaging, which shows areas of low signal intensity that co-localize with spots of strong TPEF (Fig. 3C, arrows).

The ex vivo characterization of the irradiation-induced increase in endogenous fluorescence indicated a cumulative photodamage, as reported earlier [37]. At fixed irradiance, it depended only on the total energy delivered to the sample's volume, i. e. only on the number of scans using constant acquisition parameters. For regular short term imaging we did not observe any measurable photodamage. The threshold for photodamage in our imaging system was at least tenfold higher than required for optimal imaging: At laser power of 52 mW, the first signs of photodamage on brain cryosections were usually observed after 60–100 scans (see Fig. 3), while high quality images are acquired at this power level with averaging of 4 to 8 scans.

**Figure 4:**
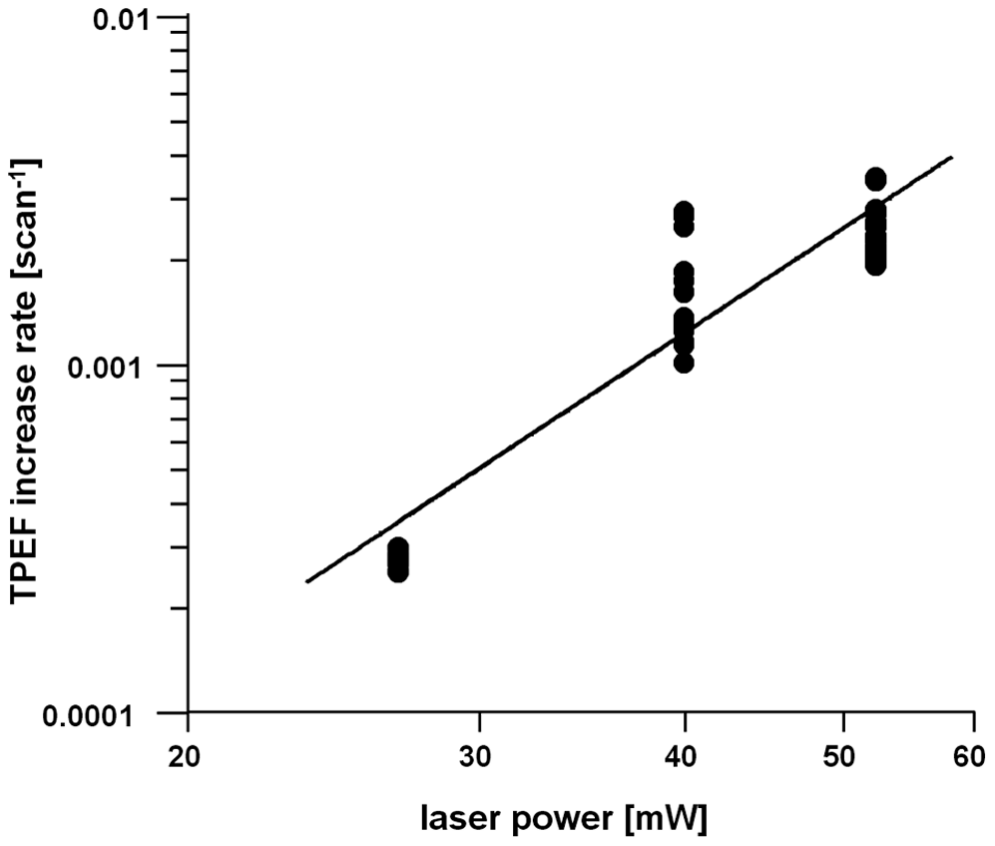
Dependence of the TPEF increase rate on laser power. The rate was obtained as inverse of the number of scans required to attain an increase of TPEF of 25% above baseline. The best fit line has a slope of 3.17±0.24 on a logarithmic scale.

The damage threshold (expressed as number of repeated scans) vs. laser power was found to be strongly nonlinear (Fig. S1). When an index for entity of photodamage is obtained as TPEF increase rate (e.g. reciprocal of the scan number required for attaining an increase of 25% over baseline), then the best fit line has a slope of 3.17±0.24 on a logarithmic scale (Fig. 4). This indicates that high order photochemical effects are involved in photodamage and is consistent with the existing bibliography, where slopes comprised between 2 [21] and 4 [38] were retrieved. Due to this nonlinear dependence, at half of the laser power (e.g. 27 mW) a very prolonged scanning is required to observe increase of TPEF (roughly 8-fold number of scans), enabling in fact to operate in the everyday use without ever observing the phenomenon. For laser power below 20 mW we did not observe increase of TPEF on brain cryosections even after 10,000 scans. It was already reported that the threshold for photodamage in terms of laser power is roughly two-fold the power required for imaging [39] and therefore our results substantially agree with the existing knowledge.

**Figure 5:**
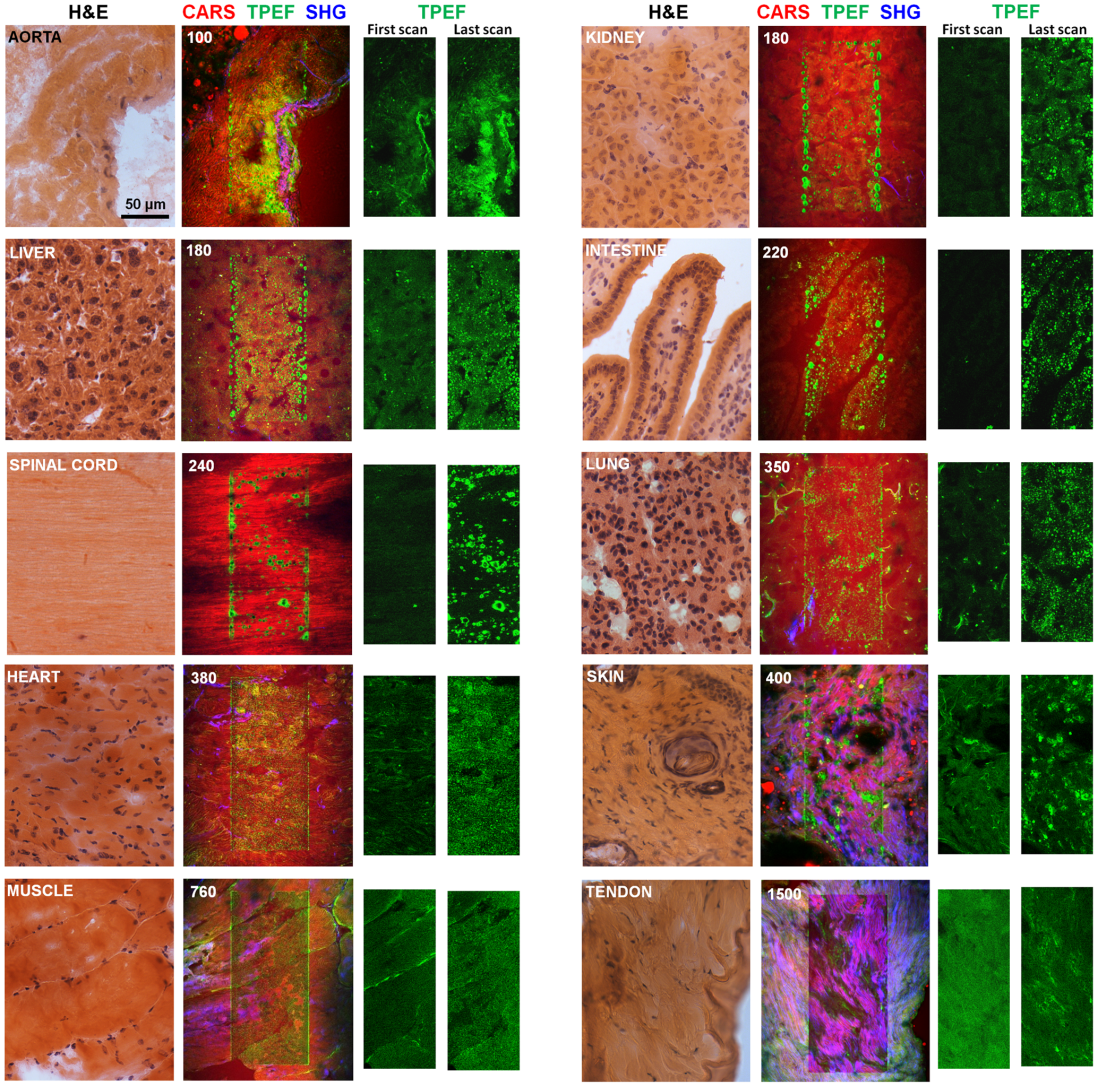
Photodamage on rehydrated cryosections of different mouse tissue types. The repetitive irradiation was performed in the central part of the images. CARS (red), TPEF (green) and SHG (blue) signals were acquired. The total number of scans used to produce the damage is also reported. The TPEF images of the first and last scan are shown for comparison. All irradiations were performed using the same experimental settings and therefore the scan numbers can be compared. Histological H&E staining of consecutive sections is reported as reference to visualize the tissue structures. All experiments were performed with a total laser power of ∼52 mW in the sample.

Further experiments were performed on cryosections of mouse nervous tissue, liver, kidney, gut, lung, aorta, heart, muscle and skin. These demonstrated that the increase of endogenous fluorescence due to prolonged irradiation is a common feature among several tissue types (Fig. 5), but the number of scans required to produce an increase in TPEF varied among the different tissue types.

The most sensitive tissue was found to be aorta, which displayed a strong irradiation-induced increase of endogenous TPEF after 100 scans only. The pattern of TPEF is related to the tissue layers composing the vessel: the *tunica intima* with the elastin lamina and the *adventitia* were characterized by an increase of TPEF, while the muscular layer of the *tunica media* rich in collagen (recognizable from the strong SHG signal) did not display appreciable increase of TPEF. Kidney, liver, intestine and spinal cord show similar behaviors; in particular the observed increase of TPEF on spinal cord is fully comparable to brain. Lung, heart and skin required a higher number of scans, but the final pattern of irradiation-induced TPEF is qualitatively the same as for the already mentioned organs. Muscle and tendon did not show a strong increase of fluorescence even after prolonged irradiation (see Fig. S2). For instance, repeated scanning of tendon was performed until 1500 scans producing mainly bleaching of the initial fluorescence. A local and weak increase of fluorescence above the level attained after bleaching was sometimes observed in the case of tendon (as in the case reported in Fig. S2), but was not consistent among our experiments. It has to be noted that a strong increase of fluorescence was instead observed in case of photomodification of collagen fibers with femtosecond lasers [40], [41] and therefore our result cannot be generalized, as damage mechanisms might change with pulse duration.

On all samples photobleaching of the initial TPEF could be observed. The effect is weak on nervous tissue (compare Fig. S1), which is characterized by a very low endogenous fluorescence, and more pronounced in tissue like skin, muscle and tendon (see Fig. S2). In all cases except tendon, the photodamage increased the local intensity of TPEF above the starting level. Nevertheless, photobleaching of the initial TPEF should be considered, as it can lead to underestimation of the photodamage thresholds.

**Figure 6:**
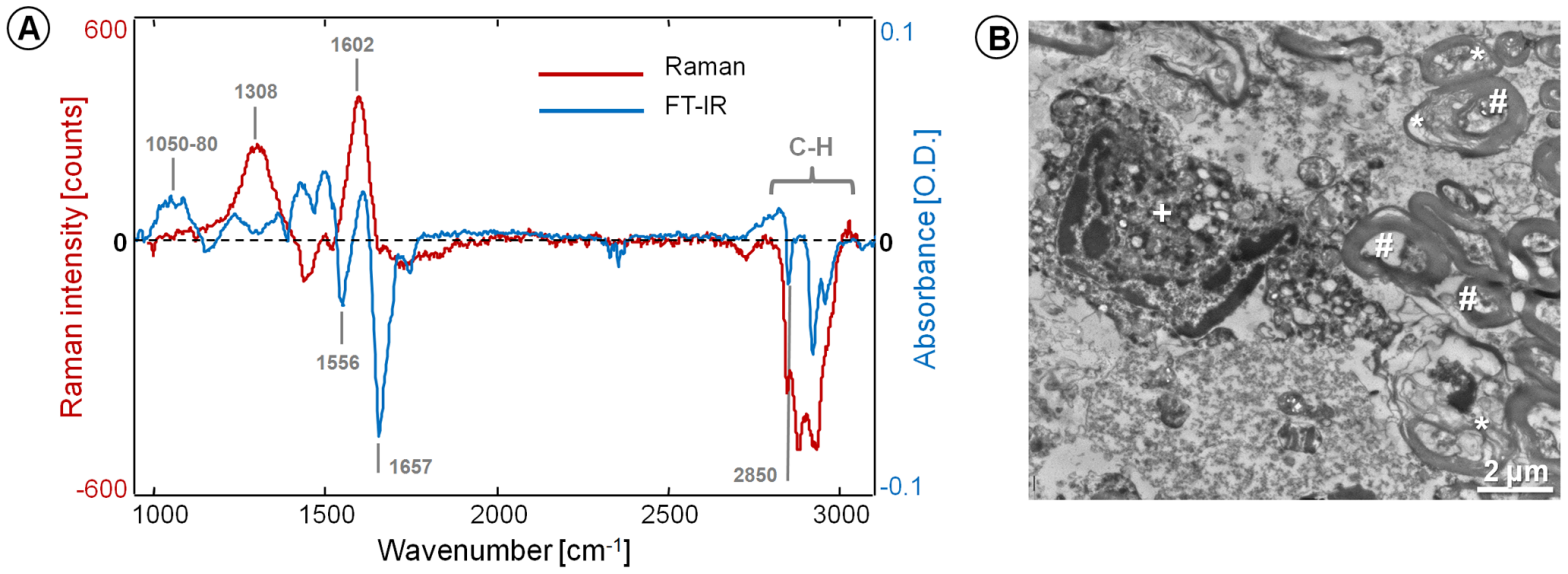
Characterization of tissue alterations connected to the irradiation-induced fluorescence. **A**: Raman and FT-IR spectra of an irradiated mouse brain cortex showing increased endogenous fluorescence. The spectra were obtained by subtraction of the not irradiated tissue spectrum from the irradiated one. The most pronounced positive and negative bands are labeled and discussed in the text. **B**: Transmission electron microscopy of an irradiated sample of human brain tissue (white matter), showing partially destroyed myelin sheaths (*), a partly damaged nucleus (+) and preserved axons (#).

Infrared and Raman spectroscopic measurements (Fig. 6A) were performed on mouse brain cryosections that displayed irradiation-induced fluorescence. Both spectroscopic methods revealed a decrease of lipid content inside the irradiated tissue, as indicated by negative bands in the high energy region of the spectrum between 2800–3000 cm^−1^ (C–H_x_ stretching in methylene and methyl molecular groups [42], [43]). The decrease of the Raman band intensity at 2850 cm^−1^, assigned to C–H symmetric stretching in methylene, confirms the observed decrease of the CARS signal intensity in the damaged area. These observations suggest that lipids are somehow “consumed” during the formation of fluorescence compounds. On the other side, a decrease in amide I and II bands [42] at 1657 and 1556 cm^−1^ respectively in the FT-IR spectra indicates a reaction that involves the amine groups of proteins. The spectral region related to nucleic acids vibrations between 1050 and 1080 cm^−1^ (symmetric stretching of phosphate groups and ribose vibration in DNA and RNA backbone [42]) did not show any appreciable variation. Other biochemical changes induced in the tissue by prolonged irradiation leading to intense photodamage-induced TPEF increase are characterized by positive bands at 1308 cm^−1^ and 1602 cm^−1^ in the Raman difference spectra, which can be assigned to the D and G bands of carbons and indicate the formation of graphite [44]. Additionally to single-point measurements, also Raman mapping experiments were performed on irradiated mouse brain tissue. Comparison with NLO microscopy proved that cluster analysis enables to distinguish between damaged and preserved tissue areas (Fig. S3A), while the analysis of the centroid spectra (Fig. S3B) indicates increase of graphite bands and decrease of the C-H_x_ stretching bands. High intensity of carbon bands was found to be a characteristic of highly fluorescent, heavily irradiated regions (Fig. S3C, 220 scans), but a significant increase in endogenous TPEF could be observed before the formation of detectable quantities of carbon (Fig. S3C, 100 scans). Therefore, the formation of carbon represents the last step in the photodamage process.

Damaged tissue areas were characterized by dark spots indicative of carbon particles that correlate with the pattern of damage-related fluorescence (Fig. S4). The black spots were detectable after H&E staining of damaged tissue. Their pattern did not reveal any correlation of the damage to a certain cell compartment. In order to link irradiation-induced fluorescence to ultrastructural damage we performed transmission electron microscopy on irradiated human brain samples. In the center of the photodamage-induced fluorescent spots the structure of white matter tissue was completely lost and carbon particles with dimension up to 0.5 µm were observed (Fig. S5). In the rim areas of the spots strongly damaged myelin sheaths, as well as partly damaged axons and nuclei, were observed (Fig. 6B). Axons and nuclei appeared to be more resistant to damage than myelin sheaths. Mitochondria remained stable, but lost their internal structure.

## Discussion

The observed increase in tissue fluorescence after pulsed NIR-laser irradiation of cells and tissue is consistent with earlier findings: An enhancement of endogenous fluorescence was detected during irradiation of melanoma and of blood cells [31]. A fluorescent scar was reported after controlled laser damaging of living cells [29]. An increase of basal fluorescence indicative of photodamage was observed during investigation of cortical neurons using different fluorescent dyes [37], on dendritic spines [32] and on branches of pyramidal neurons [33]. Other studies reported the appearance of increased luminescence during multiphoton imaging of a wing of drosophila [45] and of a short-lived luminescence in hamster ovary cells at laser power of 10 mW [46]. In these publications the source of the increase of TPEF is not addressed.

In the work of other groups the photodamage-related increase of endogenous fluorescence was attributed to NADH [34], [35], while other studies related the onset of TPEF increase to NADH bleaching, hypothesizing that this triggers photodamage by formation of reactive oxygen species [47]. Our results also suggest that a photochemical reaction is rather involved than a physiological response of the cell, as we found it also on frozen tissue sections. Additionally, the punctuated fluorescence pattern is not consistent with the diffuse increase of fluorescence in the cell cytoplasm caused by NADH [34].

The increase of TPEF could be detected in a wide spectral range from red (e.g. by using a BP filter 570–610 nm for signal detection) to blue (e.g. by using a LP filter for detection of wavelength shorter than 485 nm). The photodamage induced TPEF is not observed in the CARS channel (e.g. using a BP filter 619–675 nm) and in the SHG channel (e.g. using a BP filter 381–399 nm). This is in substantial agreement with the spectral analysis of fluorescence induced by multiphoton imaging of small intestine tissue with femtosecond laser excitation [39]. Moreover, the damage-related TPEF could also be detected with conventional fluorescence both in experiments performed on rehydrated brain sections and in vivo. We also observed that the fluorescence signal is still detectable after fixation and staining of the samples (Fig. S6), supporting the idea that stable fluorescent compounds are generated by tissue irradiation.

The fluorescence increase induced by photodamage is a process common to in vivo, fresh and ex vivo tissue. The damage threshold of nervous tissue is similar to the one found on ex vivo bulk tissue and on rehydrated cryosections (compare Fig. 1 and 3); moreover the onset of increase of fluorescence in vivo is not lower than on the same tissue ex vivo. Therefore rehydrated cryosections constitute an inexpensive and easy–to–use model to assess the tissue specific sensitivity and choose safe parameters for in vivo imaging.

The decrease in the CARS signal and the results of FT-IR and Raman spectroscopy indicate that the formation of different fluorescent compounds inside the tissue is accompanied by a decrease of lipid and protein content. In the experiments performed on different tissue types, a lower photodamage-induced fluorescence was obtained on lipid-poor samples as heart and muscle; no evident increase of fluorescence was registered even after a very long scanning on tendon, which predominantly constitutes of extracellular matrix proteins.

These observations suggest a direct involvement of tissue lipids in the photochemical processes underlying TPEF increase. It is well known that the interaction of unsaturated lipids with UV and visible radiation leads to peroxidation in presence of endogenous sensitizers such as NAD(P)H and flavines [48]. Lipid peroxidation produces highly reactive compounds that further crosslink with other molecules in the tissue such as amines to produce fluorescent compounds like Schiff's bases and/or dihydropyridine compounds [49], [50]. Considering that a photo-oxidation can be induced by one- and two-photon absorption mechanisms during laser irradiation, we hypothesize that such kind of reactions underlies the observed increase in endogenous fluorescence.

We did not observe any increase of fluorescence inside the cell nucleus (see Fig. 1, 2 and 3B), nor find any evidence of two-photon damage involving nucleic acids from spectroscopy. The absorption of DNA is limited to the region below 300 nm [51] and this would imply a three-photon process at an irradiation wavelength of 780 nm.

The availability of an intrinsic indicator of photodamage arising during multiphoton experiments is valuable whenever the laser power inside the sample is higher than 10 mW, which is a power normally considered safe for imaging [4], [12], [14] but not sufficient for high-speed imaging using endogenous signals such as CARS. At laser power above this value, the photodamage threshold depends on the specific system configuration (laser wavelength, laser pulse duration, power density inside the sample) and, even across the same sample, the damage arises heterogeneously, reflecting local differences in tissue absorption. This variability makes a priori estimates of photodamage threshold difficult. Therefore, monitoring the increase of fluorescence by acquisition of TPEF is very useful for the detection of irradiation-induced tissue damage in real-time.

Photodamage has been investigated in the past by several groups, which used different experimental setups and biological models. Different laser wavelength and laser pulse length are commonly used for multiphoton microscopy and were also employed for characterization of photodamage. A possibility to compare different experiments with similar laser wavelength (near infrared, 700–1000 nm) is the calculation of the irradiance I =  E_pulse_/τA, where E_pulse_ is the laser pulse energy, τ the pulse length and A the spot area in the laser focus calculated as Airy disk. It is then possible to find different degrees of photodamage in a wide irradiance range: from I = 0.06 ⋅ 10^12^ W/cm^2^ (cell blebbing, myelin sheet damage [20]) and I = 0.2 ⋅ 10^12^ W/cm^2^ (morphological cell damage [28]), up to I = 1 ⋅ 10^12^ W/cm^2^ (chromosome dissection [52]). In our experimental setup the irradiance required for fast and high quality imaging ranges from I = 0.07 ⋅ 10^12^ W/cm^2^ (27 mW in the laser focus) to I = 0.13 ⋅ 10^12^ W/cm^2^ (52 mW in the laser focus). These values are comparable with the irradiance used in photodamage experiments already reported. The irradiance values also provide an insight in the potential mechanisms underlying the observed photodamage. From theoretical simulations of the interaction between ultrashort laser pulses and homogeneous transparent media [52], the irradiance threshold I_b_ for optical breakdown can be retrieved. For 1 ps laser pulses I_b_≅2 ⋅ 10^12^ W/cm^2^, which is much higher than the highest irradiance used in our experiments. The threshold for formation of low density plasma was estimated by Vogel et al. [52] to be 0.04 ⋅ I_b_, e.g. in our case I≅0,08 ⋅ 10^12^ W/cm^2^. Therefore formation of free electrons in the biological materials is expected in all experiments performed with a laser power of 52 mW and may contribute considerably to the observed damage. Moreover, photodamage could be mediated by several endogenous tissue pigments and chromophores, which absorb the laser radiation in linear and nonlinear processes [53], [54].

The irradiance threshold for cell death at 80 MHz repetition rate was estimated [52] to be 0.067 ⋅ I_b_, which is very close to the irradiance obtained in our system with a laser power of 52 mW. This justifies the heavy damage observed during irradiation of living glioblastoma cells.

Besides increase of TPEF, the formation of carbon particles is a further characteristic of heavy photodamage. The formation of graphite could be caused by free-electron-mediated tissue disintegration as reported in [52]. The creation of carbon particles possibly triggers additional thermal damage by one-photon absorption. As the irradiance used in the experiments is well below the one required for water breakdown and for heating at boiling temperature (estimated at 0.5 ⋅ I_b_ [52]), we hypothesize that the long-lived bubbles often observed at the very last stage of repetitive scanning are in fact produced by water and/or tissue vaporization due to overheating on carbon particles. Formation of low density plasma and subsequent overheating of carbon particles could also justify the heavy damage to tissue structures as visualized by electron microscopy (Fig. S5).

In summary, we provide a new insight in multiphoton induced damage, demonstrating that at the high laser power required for label-free multiphoton microscopy there is a second photomechanism of damage leading to increased tissue fluorescence, which is different from the already observed increase of cellular NADH. For this type of photodamage, which is due to photochemical effects independent from physiological cell responses, the use of rehydrated cryosection constitutes a model to set safe imaging parameters with any experimental configuration prior in vivo imaging in order to protect cells and tissues.

## Supporting Information

Figure S1 Physical characterization of endogenous TPEF induced by serial irradiation on rehydrated cryosections of mouse cortical tissue. The intensity of TPEF signal was measured in 13 regions of interest (Roi) during the serial scanning of brain tissue. **A**: The location of Rois is illustrated in TPEF images of brain tissue at after 10, 500 and 800 scans (thin blue lines). **B**: The corresponding mean fluorescence intensity of Roi A (indicated in gray in A) and Roi B (indicated in white in A) for the time course of the experiment is shown. Baseline values were calculated after initial bleaching using the average of 10 TPEF-intensity values (indicated in blue). An increase in TPEF signal of +25% above the baseline was considered as onset of photo damage. The corresponding number of scans for each Roi was used to calculate an average onset of photo damage for the sample. The number of scans needed to induce photodamage in this sample was 310 for Roi A (red arrow) and 530 for Roi B (orange arrow). **C**: Plot of the number of scans (median and range) needed to cause photo damage vs. laser power; the relationship is strongly non linear. Experiments were done with constant system configuration. After replacement of optical components and required realignment we noticed an increased over-all damaging capacity at the same power levels compared to the data shown here (compare also Fig. 3 and Fig. S3C).
(TIF)

Figure S2 Local variation of TPEF and CARS intensity during repetitive scanning of skin, muscle and tendon cryosections. TPEF and CARS images selected at the beginning, after bleaching and at the maximum of subsequent TPEF increase are shown, together with the plot of the normalized intensity measured in the area identified by the boxes as function of the number of scans. In all cases a strong bleaching of the initial fluorescence was first observed, followed by simultaneous increase of TPEF and decrease of CARS intensities. In the case of skin the intensity of photodamage-induced TPEF increases well above the starting value. In the cases of muscle, after the initial bleaching it increases up to a value that is close to the starting one. In the case of tendon, no increase of TPEF could be observed in most of the irradiated area, but a weak local increase, followed by further bleaching, characterizes the area in the box used for the quantification. The scanning sequences analyzed in this picture are the same whose effects are shown in Fig. 5.
(TIF)

Figure S3 Raman mapping of irradiated mouse brain sections. **A**: map obtained by clustering of the Raman spectra in two clusters (blue: not irradiated and preserved tissue; red: damaged tissue), overlaid with the CARS image (gray scale) that indicates regions of severe photo damage (dark spots: loss in CARS signal, compare Fig. 3). **B**: Centroid spectra of the cluster map shown in A (blue: not irradiated and preserved tissue; red: damaged tissue). **C**: intensity of the G Raman band of carbon at 1602 cm^−1^ calculated from a line map across areas irradiated with different parameters and shown in the TPEF picture. The position of the line map is indicated by the white dotted line. The increase of the intensity of the G band of carbon is related to strong damage only. Inside the area irradiated with 220 scans the highest integral intensity of the G band was measured. In the area irradiated with 100 scans, an increase of the G band intensity was detected only in the left part (where the increase in TPEF was measured to be +280%); in the right part, no significant increase of Raman intensity was observed, while the average increase of TPEF was measured to be +80%. In the area irradiated with 75 scans, the TPEF increase over background was found to be around 15% and no increase of the G band associated with carbon compounds could be detected.
(TIF)

Figure S4 Photodamage on mouse brain cortical tissue: correlation with histological staining. **A**: TPEF (green) and CARS (red) image acquired on rehydrated mouse brain tissue immediately after 950 scans of the area showing strong TPEF. **B**: H&E staining of the sample shown in A. Dark spots were detected in the irradiated area. **C**: overlay of TPEF image shown in A and H&E image shown in B. **D**: Magnification of the area indicated in C, revealing a similar pattern of the TPEF signal (indicative of photodamage) and the dark spots.
(TIF)

Figure S5 Transmission electron microscopy picture of a photodamage-induced fluorescent spot on human white matter. **A**: In the center of the spot the white matter is totally disrupted with appearance of electron-dense carbon particles (arrowhead). In the rim area of the spot, partly damaged myelin sheaths and axon fibers can be recognized. B: Detail of the carbon particle indicated by the arrowhead in the panel A.
(TIF)

Figure S6 Immunohistochemistry of a mouse brain cryosection with photodamage-induced fluorescence. The pattern of TPEF induced by repetitive scanning on the cortical region (shown on the right) can be retrieved by conventional fluorescence excited with mercury lamp after tissue processing (compare with the green pattern on the left picture; blue: DAPI, red: GFAP).
(TIF)

Video S1 Video of mouse brain cortex during 5 minutes of repetitive scanning in vivo. (also shown in Fig. 2C, D). The blood flowing in the blood vessel can be seen until the final phase of the experiment; a progressive increase of photodamage-induced TPEF can be observed in several spots.
(AVI)

Video S2 Increase of TPEF during 10 minutes of serial scanning on a rehydrated cryosection of mouse cortical tissue.
(AVI)
