# Supplementary figures and images for: Intrinsic Indicator of Photodamage during Label-Free Multiphoton Microscopy of Cells and Tissues
Source: PLoS One. 2014 Oct 24;9(10):e110295. doi: 10.1371/journal.pone.0110295 (PMC4208781; doi:10.1371/journal.pone.0110295)

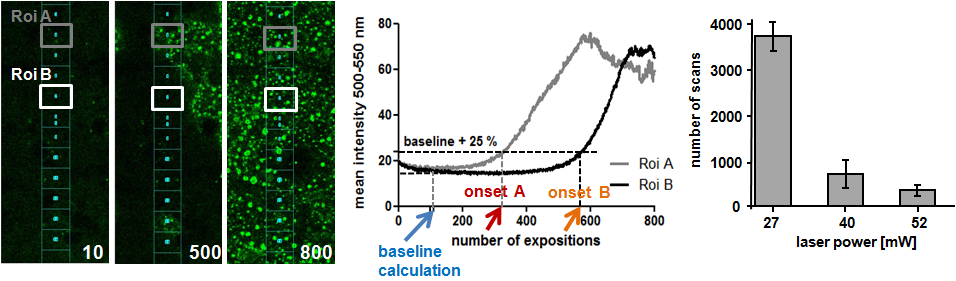

Supplement: Figure S1 — Physical characterization of endogenous TPEF induced by serial irradiation on rehydrated cryosections of mouse cortical tissue. The intensity of TPEF signal was measured in 13 regions of interest (Roi) during the serial scanning of brain tissue. A: The location of Rois is illustrated in TPEF images of brain tissue at after 10, 500 and 800 scans (thin blue lines). B: The corresponding mean fluorescence intensity of Roi A (indicated in gray in A) and Roi B (indicated in white in A) for the time course of the experiment is shown. Baseline values were calculated after initial bleaching using the average of 10 TPEF-intensity values (indicated in blue). An increase in TPEF signal of +25% above the baseline was considered as onset of photo damage. The corresponding number of scans for each Roi was used to calculate an average onset of photo damage for the sample. The number of scans needed to induce photodamage in this sample was 310 for Roi A (red arrow) and 530 for Roi B (orange arrow). C: Plot of the number of scans (median and range) needed to cause photo damage vs. laser power; the relationship is strongly non linear. Experiments were done with constant system configuration. After replacement of optical components and required realignment we noticed an increased over-all damaging capacity at the same power levels compared to the data shown here (compare also Fig. 3 and Fig. S3C). (TIF) [file pone.0110295.s001.tif]

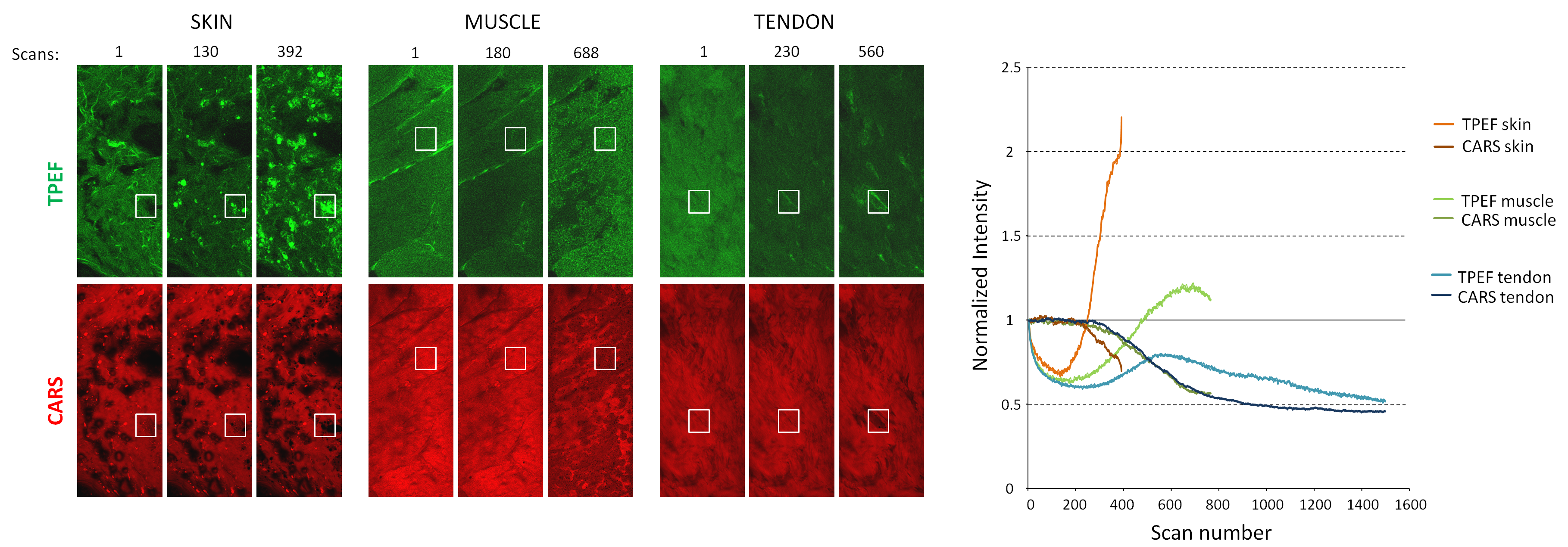

Supplement: Figure S2 — Local variation of TPEF and CARS intensity during repetitive scanning of skin, muscle and tendon cryosections. TPEF and CARS images selected at the beginning, after bleaching and at the maximum of subsequent TPEF increase are shown, together with the plot of the normalized intensity measured in the area identified by the boxes as function of the number of scans. In all cases a strong bleaching of the initial fluorescence was first observed, followed by simultaneous increase of TPEF and decrease of CARS intensities. In the case of skin the intensity of photodamage-induced TPEF increases well above the starting value. In the cases of muscle, after the initial bleaching it increases up to a value that is close to the starting one. In the case of tendon, no increase of TPEF could be observed in most of the irradiated area, but a weak local increase, followed by further bleaching, characterizes the area in the box used for the quantification. The scanning sequences analyzed in this picture are the same whose effects are shown in Fig. 5. (TIF) [file pone.0110295.s002.tif]

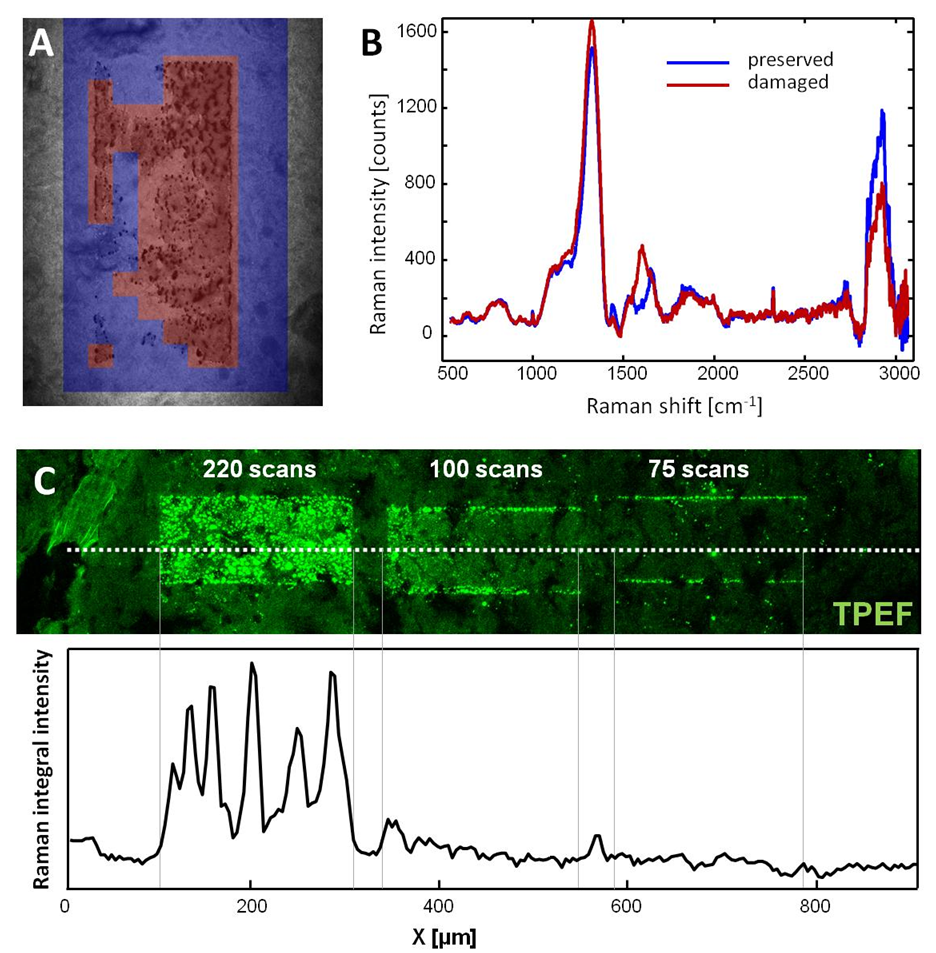

Supplement: Figure S3 — Raman mapping of irradiated mouse brain sections. A: map obtained by clustering of the Raman spectra in two clusters (blue: not irradiated and preserved tissue; red: damaged tissue), overlaid with the CARS image (gray scale) that indicates regions of severe photo damage (dark spots: loss in CARS signal, compare Fig. 3). B: Centroid spectra of the cluster map shown in A (blue: not irradiated and preserved tissue; red: damaged tissue). C: intensity of the G Raman band of carbon at 1602 cm−1 calculated from a line map across areas irradiated with different parameters and shown in the TPEF picture. The position of the line map is indicated by the white dotted line. The increase of the intensity of the G band of carbon is related to strong damage only. Inside the area irradiated with 220 scans the highest integral intensity of the G band was measured. In the area irradiated with 100 scans, an increase of the G band intensity was detected only in the left part (where the increase in TPEF was measured to be +280%); in the right part, no significant increase of Raman intensity was observed, while the average increase of TPEF was measured to be +80%. In the area irradiated with 75 scans, the TPEF increase over background was found to be around 15% and no increase of the G band associated with carbon compounds could be detected. (TIF) [file pone.0110295.s003.tif]

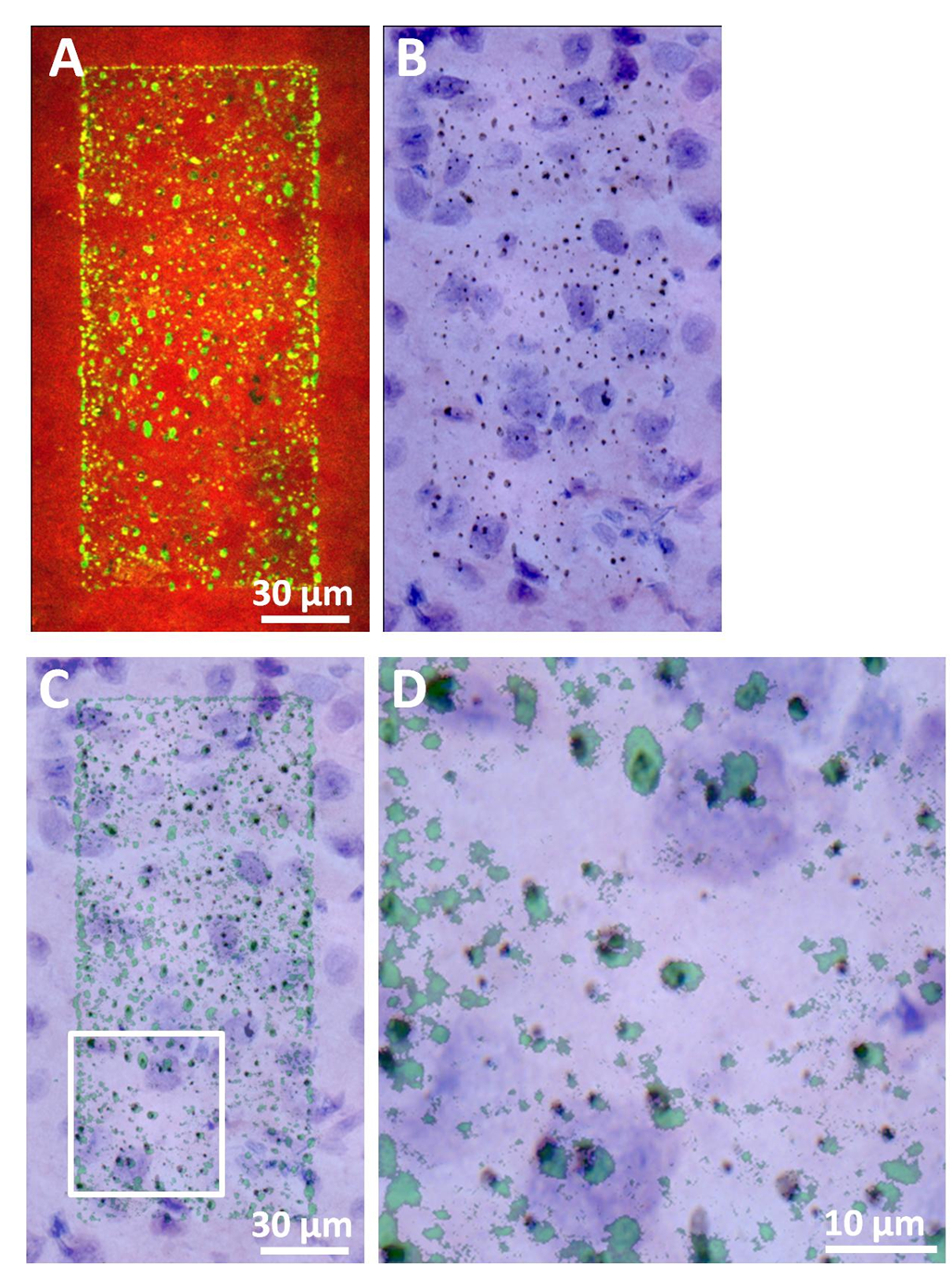

Supplement: Figure S4 — Photodamage on mouse brain cortical tissue: correlation with histological staining. A: TPEF (green) and CARS (red) image acquired on rehydrated mouse brain tissue immediately after 950 scans of the area showing strong TPEF. B: H&E staining of the sample shown in A. Dark spots were detected in the irradiated area. C: overlay of TPEF image shown in A and H&E image shown in B. D: Magnification of the area indicated in C, revealing a similar pattern of the TPEF signal (indicative of photodamage) and the dark spots. (TIF) [file pone.0110295.s004.tif]

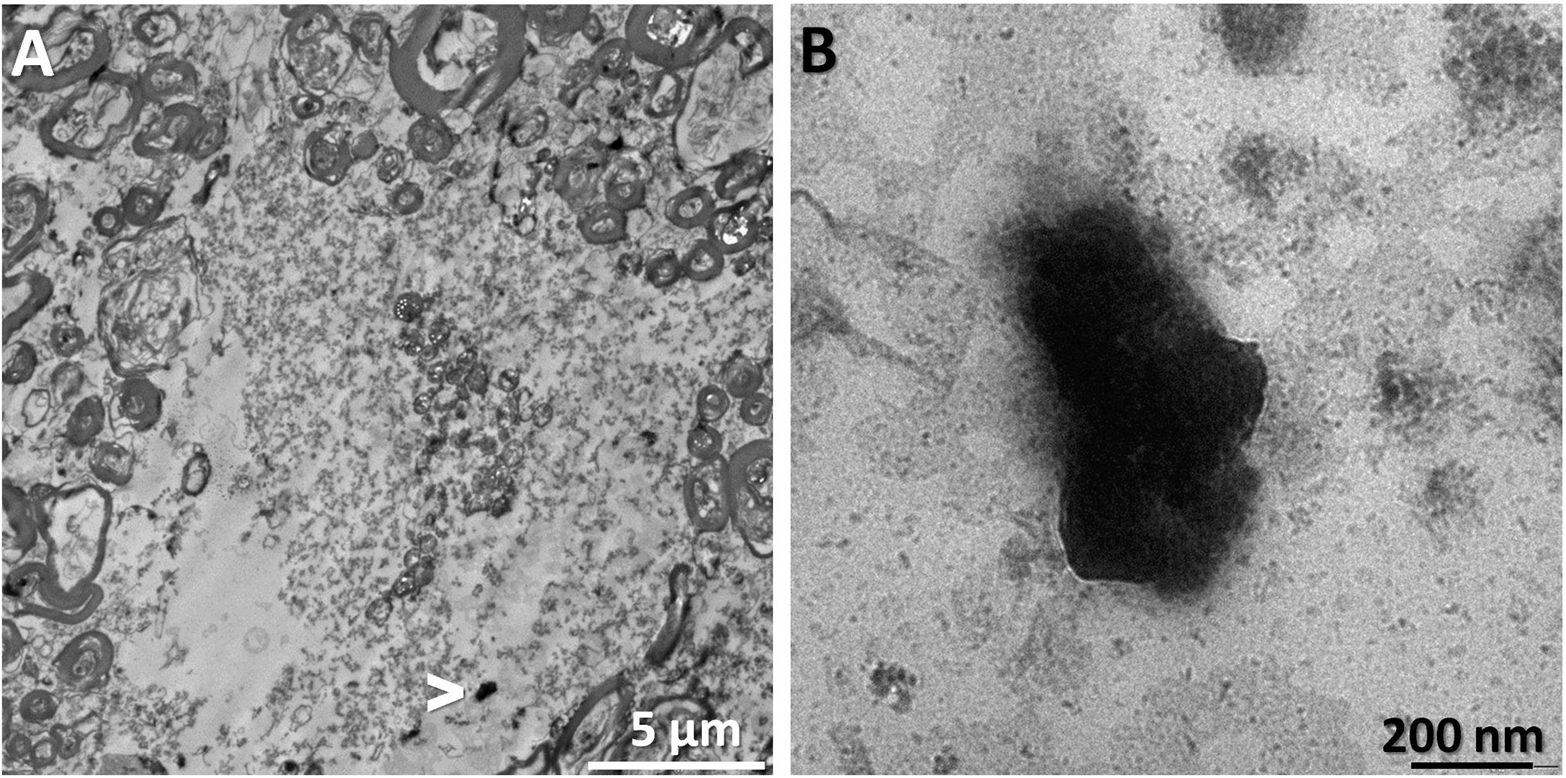

Supplement: Figure S5 — Transmission electron microscopy picture of a photodamage-induced fluorescent spot on human white matter. A: In the center of the spot the white matter is totally disrupted with appearance of electron-dense carbon particles (arrowhead). In the rim area of the spot, partly damaged myelin sheaths and axon fibers can be recognized. B: Detail of the carbon particle indicated by the arrowhead in the panel A. (TIF) [file pone.0110295.s005.tif]

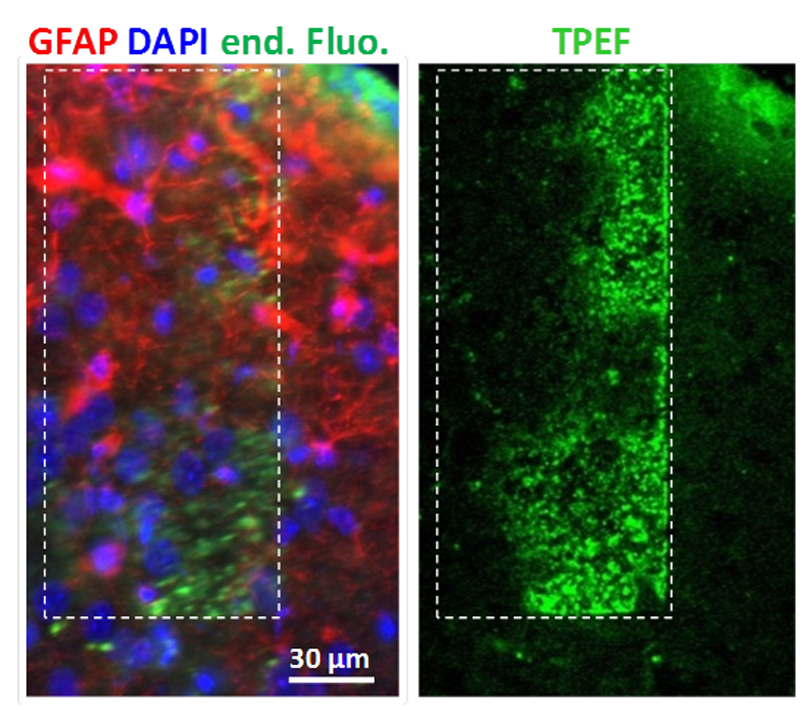

Supplement: Figure S6 — Immunohistochemistry of a mouse brain cryosection with photodamage-induced fluorescence. The pattern of TPEF induced by repetitive scanning on the cortical region (shown on the right) can be retrieved by conventional fluorescence excited with mercury lamp after tissue processing (compare with the green pattern on the left picture; blue: DAPI, red: GFAP). (TIF) [file pone.0110295.s006.tif]
